# Fatal *Balamuthia mandrillaris* Meningoencephalitis in the Netherlands after Travel to The Gambia 

**DOI:** 10.3201/eid2105.141325

**Published:** 2015-05

**Authors:** Nadine A.M.E. van der Beek, Carla van Tienen, Jubi E. de Haan, Jeroen Roelfsema, Pieter J. Wismans, Perry J.J. van Genderen, Herve L. Tanghe, Rob M. Verdijk, Maarten J. Titulaer, Jaap J. van Hellemond

**Affiliations:** Erasmus University Medical Centre, Rotterdam, the Netherlands (N.A.M.E. van der Beek, C. van Tienen, J.E. de Haan, H.L. Tanghe, R.M. Verdijk, M.J. Titulaer, J.J. van Hellemond);; National Institute for Public Health and the Environment, Bilthoven, the Netherlands (J. Roelfsema);; Harbor Hospital, Rotterdam (P.J. Wismans, P.J.J. van Genderen, J.J. van Hellemond)

**Keywords:** encephalitis, meningoencephalitis, free-living ameba, *Balamuthia mandrillaris*, parasites, protozoa, meningitis, The Gambia, the Netherlands

**To the Editor:** *Balamuthia mandrillaris* is a free-living ameba that has a worldwide distribution in soil and was first reported in 1990 (*1*). Approximately 200 *B. mandrillaris* meningoencephalitis cases have been described, mostly from warm climate areas in South America. Its prevalence in the United States is estimated to be 1 case/year (*2*). However, *B. mandrillaris* meningoencephalitis has not been reported in Africa, and only 4 cases have been reported in Europe (*3*–*6*). Transmission occurs through the respiratory tract or the skin or by organ transplant, and the incubation period varies from weeks to months after primary infection (*7*). After an indolent, subacute phase with aspecific symptoms, the amebae invade the central nervous system, and illness rapidly progresses, leading almost invariably to death (*7*). Because *B. mandrillaris* is difficult to detect in soil, its specific geographic distribution around the world is unknown and is estimated on the basis of where illnesses have been reported (*7*). This report addresses fatal *B. mandrillaris* meningoencephalitis in a woman from the Netherlands who had visited The Gambia.

In December 2013, a previously healthy 61-year-old white woman in the Netherlands sought care for fever, headaches, and muscle pains she had experienced for 1 week. That year, she had traveled 4 times to The Gambia, the last visit being 1 month before her hospitalization (Technical Appendix Table 1). After she returned from her visit in September 2013, fatigue, diarrhea, fever, and pustular skin lesions on her back and lower extremities developed. A wound swab culture showed *Staphylococcus aureus*, for which she was treated successfully with oral clarithromycin and topical fucidin ointment.

On admission in December, her physical and neurologic examination results were unremarkable. Malaria was excluded; because of persisting headaches, a cerebral computed tomography scan without contrast was performed but showed no abnormalities. In the following days, high fevers, altered mental status, and nuchal rigidity without focal neurologic deficits developed. Cerebrospinal fluid (CSF) examination showed mononuclear pleocytosis, highly elevated protein levels, and low glucose levels (Technical Appendix Table 2). Serial cerebral computed tomography and magnetic resonance imaging scans showed development of an asymmetric hydrocephalus and diffuse leptomeningeal and subependymal contrast enhancement, especially around the brainstem, without signs of intracerebral mass lesions (Technical Appendix Figure).

Presumed diagnosis was tuberculous meningitis, and she was treated with tuberculostatic drugs (isoniazid, rifampin, pyrazinamide, and ethambutol) combined with intravenous acyclovir, ceftriaxone, and co-trimoxazole for other infectious causes of meningoencephalitis. Despite external lumbar and ventricular (both lateral ventricles and fourth ventricle) CSF drainage, her neurologic condition deteriorated. Multiple cranial nerve palsies developed, and she became comatose and died 11 days after admission.

Informed consent for postmortem examination was obtained, and macroscopic pathologic examination showed uncal and cerebellar herniation caused by increased intracranial pressure. Microscopic brain tissue examination showed signs of acute granulomatous inflammation, multiple hemorrhagic infarctions, and angiitis in the presence of numerous amebic trophozoites and cysts (Figure), which showed granulomatous hemorrhagic necrotic amebic meningoencephalitis. Real-time PCR and subsequent sequencing on brain biopsy and CSF specimens showed *B. mandrillaris* to be the causative ameba (*8*,*9*).

**Figure:**
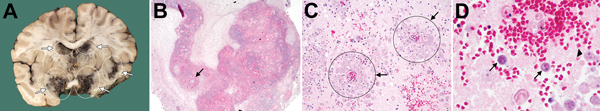
Postmortem pathologic findings for woman in the Netherlands who died of *Balamuthia mandrillaris* meningoencephalitis after returning from travel to The Gambia. A) Macroscopic coronal central section scan showing hemorrhagic necrotizing lesions of the subependymal, meningeal, and parenchymal areas of the parietotemporal lobes (circles and arrows). B) Low-power microscopic scan showing hemorrhagic necrotizing angiitis of the meningeal vessels (arrow) (original magnification ×25). C) Medium-power microscopic scan (original magnification ×200) showing perivascular trophozoite cuffing (arrows) and granulomatous inflammation. D) High-power microscopic scan (original magnification ×630) showing encysted amebae (arrows) and free trophozoites (arrowhead). Hematoxylin and eosin stains.

The infection could have been acquired in The Gambia or the Netherlands because the patient had intensive soil contact in The Gambia, where she frequently cultivated land, and in the Netherlands, where she worked in glass horticulture. She may have been infected through the skin after contact with contaminated soil, but her skin lesions were atypical for *B. mandrillaris*, and postmortem examinations failed to identify *B. mandrillaris* except in the central nervous system.

The lack of reported *B. mandrillaris* cases from Africa might indicate a low number of postmortem examinations and little access to advanced diagnostics, rather than a low environmental prevalence of *B. mandrillaris*. The few reported cases in Europe might be related to lack of awareness and to clinical signs and symptoms that mimic tuberculous meningitis: a lymphocytic pleocytosis with an elevated protein level and a low glucose level in CSF, together with a hydrocephalus and subependymal and leptomeningeal contrast enhancement on magnetic resonance imaging (*10*). Also, *B. mandrillaris* meningoencephalitis imaging findings are often nonspecific, including cerebral edema, hydrocephalus, multiple space-occupying and ring-enhancing lesions, leptomeningeal enhancement, or formation of mycotic aneurysms (*2*). Furthermore, amebic trophozoites are seldom detected in CSF by microscopy (*2*,*3*). Consequently, *B. mandrillaris* meningoencephalitis could be underdiagnosed, especially where this infection has no or only sporadic reports.

*B. mandrillaris* should be considered in refractory or unexplained cases of meningoencephalitis, even outside the Americas and in immunocompetent patients. Detecting *B. mandrillaris* by PCR in CSF seems most likely to enable early diagnosis and timely treatment. However, appropriate therapy is not well defined; success has been sparsely reported with the simultaneous use of azoles, flucytosine, pentamidine, sulfazidine, macrolide antimicrobial drugs, phenothiazines, and miltefosine (*2*,*7*,*10*).

**Technical Appendix.** Timeline of relevant events, laboratory investigations during hospitalization, and imaging results for Dutch traveler who died of *Balamuthia mandrillaris* meningoencephalitis after returning from The Gambia.
