# Supplementary material for: Fatal Balamuthia mandrillaris Meningoencephalitis in the Netherlands after Travel to The Gambia
Source: Emerg Infect Dis. 2015 May;21(5):896–8. doi: 10.3201/eid2105.141325 (PMC4412220; doi:10.3201/eid2105.141325)
Supplement: Supplementary file 1 — Technical Appendix. Timeline of relevant events, laboratory investigations during hospitalization, and imaging results for Dutch traveler who died of Balamuthia mandrillaris meningoencephalitis after returning from The Gambia. [file 14-1325-Techapp-s1.pdf]

Article DOI: http://dx.doi.org/10.3201/eid2105.141325

Fatal *Balamuthia mandrillaris* Meningoencephalitis after Travel to The Gambia, the Netherlands

Technical Appendix

Technical Appendix Table 1. Timeline of relevant events, *Balamuthia mandrillaris* meningoencephalitis patient, the Netherlands

Date	Event	Details
Feb 2013	Gambia visit	1-week visit
May 2013	Gambia visit	1-week visit
Aug 2013	Gambia visit	1-week visit
Sept 2013	First illness	Fatigue, diarrhea, fever, and pustular skin lesions on patient's back and lower extremities.<br>Wound swab culture collected from lesion showed *Staphylococcus aureas*, for which patient was successfully treated with oral clarithromycin and topical fucidine ointment.
Nov 2013	Gambia visit	1-week visit during week 1 of November
Dec 2013	Second illness	Fever and persisting headaches for 6 days; local hospital admittance in week 2 of December.<br>On day 4 after admission, patient was referred to the Rotterdam Harbour Hospital because of progressive disease. First cerebrospinal fluid sample collected.<br>On day 6 after admission, patient was referred to the Erasmus University Medical Center in Rotterdam because of progressive disease and increasing cranial pressure.<br>On day 11 after admission, patient died.
Spring 2014	Postmortem analysis	Pathologic and histologic examination of postmortem-collected brain, skin, and lung specimens. *B. mandrillaris* trophozoites and cysts were observed in brain tissue.<br>PCR analysis showed presence of *B. mandrillaris* in postmortem brain biopsy specimens and in cerebrospinal fluid specimens collected on day 4 and 7 after admission.

Technical Appendix Table 2. Laboratory investigations during hospitalization of *Balamuthia mandrillaris* meningoencephalitis patient, the Netherlands*

Investigation	Specimen	Findings
General	Blood	ESR 33 mm/h; leukocytes 17.3 10^9/L; other blood cell counts, electrolytes, liver enzymes, and kidney function tests were normal<br>B2-microglobulin, IgG1–IgG4, and paraprotein levels were within reference ranges
	CSF	Day 4: leukocytes 366 10^6/L (345 monocytes); protein 721 mg/dL; glucose 1.2 mmol/L (serum 7.1 mmol/L)<br>Day 7: leukocytes 262 10^6/L (256 monocytes); protein 1,320 mg/dL; glucose 2.0 mmol/L<br>No evidence for monoclonal B-cell population or abnormal T-cell population
Microbiology	Blood	Cultures: negative<br>Tuberculosis†: negative<br>Schistosoma‡: negative
	CSF	Malaria§: negative<br>Trypanosomiasis Gambiense¶: negative<br>Cryptococcus antigen‡: negative<br>Cultures: negative for aerobic and anaerobic bacteria and fungi<br>Mycobacteria#: negative<br>Amebae**: negative
Virology	Blood	West Nile virus, HIV, HTLV-1/2, CMV, mumps, Rift Valley fever virus†: negative<br>Rickettsia (Spotted fever group and typhoid fever group) ††: negative<br>EBV IgG VCA and NA: positive: IgM VCA†: negative<br>EBV, West Nile virus‡‡: negative

Investigation	Specimen	Findings
	CSF	HSV types 1 and 2, VZV, CMV, human herpesvirus type 6, enterovirus, West Nile virus‡‡: negative<br>EBV‡‡: positive
Pathology	CSF	No malignant cells

*ESR, erythrocyte sedimentation rate; Ig, immunoglobulin; CSF, cerebrospinal fluid; HTLV, human T-cell lymphotropic virus;, CMV, cytomegalovirus; EBV, Epstein-Barr virus; VCA, viral capsid antigen; NA, nuclear antigen; HSV, herpes simplex virus; VZV, varicella zoster virus.
†Interferon Gamma Release Assay (Quantiferon; QIAGEN, Valencia,CA, USA).
‡Serology.
§Thick blood film and PCR.
¶Quantitative buffy coat.
#PCR, auramine staining, culture.
**No trophozoites by extensive microscopic inspection.
††Immunofluorescence.
‡‡PCR.

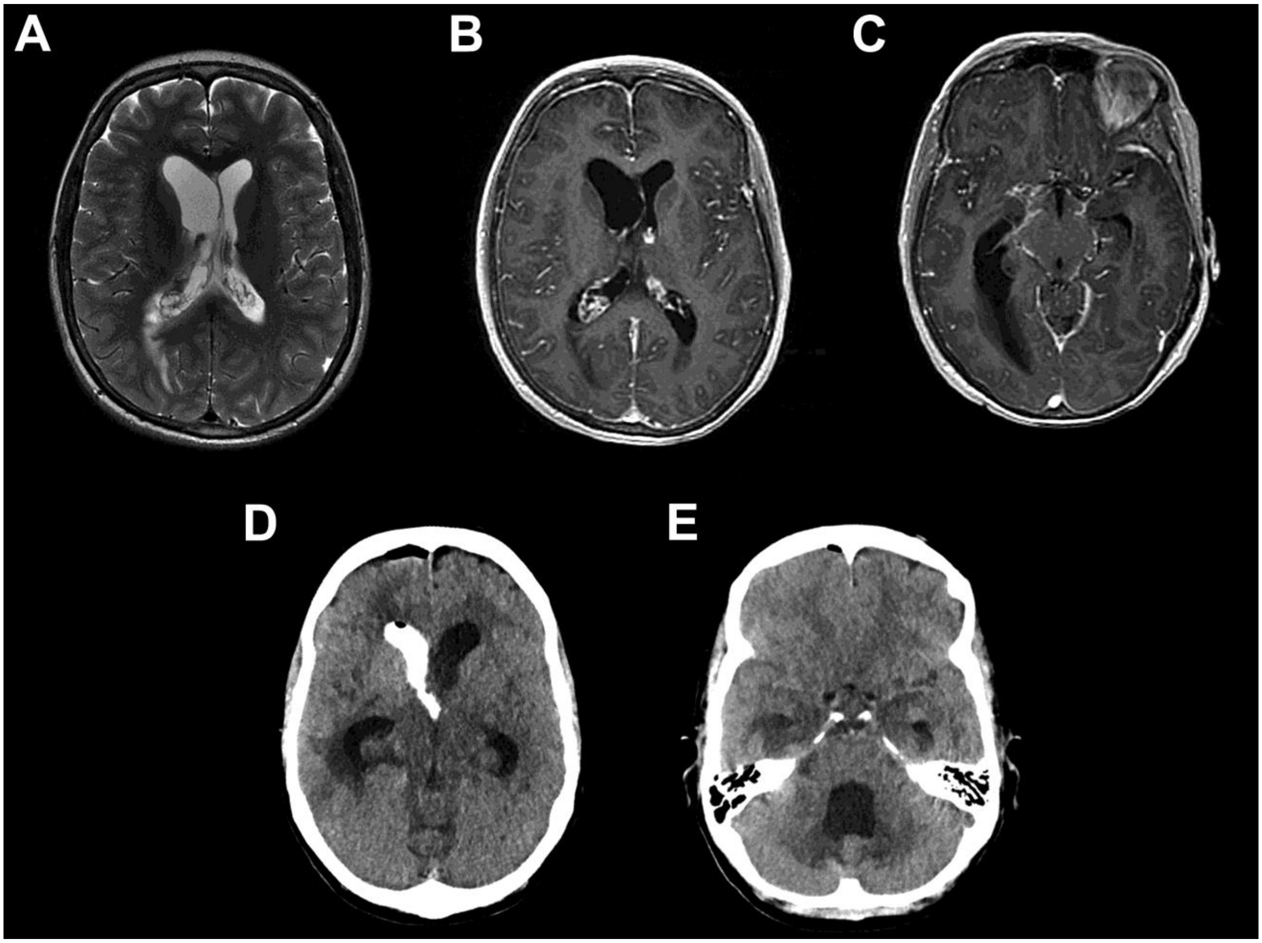

Technical Appendix Figure. Imaging findings for woman from the Netherlands who died of *Balamuthia mandrillaris* meningoencephalitis after returning from The Gambia. A) Axial MRI T2. B, C) T1 + intravenous Gadolinium. D, E) Computed tomography imaging sequences obtained from patient. A) Asymmetric hydrocephalus, which suggests compartmentalization within the ventricles because of high protein levels in the cerebrospinal fluid; enlarged plexus choroideus is also shown. B, C) Diffuse leptomeningeal and subependymal contrast enhancement without solid intracerebral mass lesions. D, E) Two hours after injection of intraventricular Visipaque contrast (GE HealthCare, Piscataway, NJ, USA), no diffusion of contrast occurred except in the right frontal horn and the third ventricle because of compartmentalization.
